# Ferroptosis and its potential role in gestational diabetes mellitus: updated evidence from pathogenesis to therapy

**DOI:** 10.3389/fendo.2023.1177547

**Published:** 2023-08-18

**Authors:** Yan Zhao, Qianqian Gao, Baoxuan Li, Yang Wang, Yue Wang

**Affiliations:** ^1^ Department of Obstetrics and Gynaecology, Shengjing Hospital of China Medical University, Shenyang, Liaoning, China; ^2^ Department of Obstetrics, Weifang People’s Hospital, Weifang, ShanDong, China

**Keywords:** gestational diabetes mellitus, ferroptosis, bioinformatic, WGCNA, molecular docking, molecular dynamic simulation

## Abstract

**Background:**

Studies have demonstrated that high iron status is positively associated with gestational diabetes mellitus (GDM), implying that iron overload and ferroptosis play important roles in the development of GDM. The aim of this study was to explore effective therapeutic drugs from traditional Chinese medicine (TCM)formulas for the treatment of GDM based on ferroptosis.

**Methods:**

In this study, the presence of ferroptosis in the placenta was verified through clinical and experimental data, and key genes were subsequently screened for association with ferroptosis in the development of GDM. The analysis was based on transcriptome sequencing of datasets combined with differentially expressed genes (DEGs) analysis and weighted gene correlation network analysis (WGCNA); functional enrichment analysis was also performed. A protein−protein interaction (PPI) network was constructed and pivotal genes were identified using Cytoscape. Finally, traditional Chinese medicine (TCM)formulas related to treating GDM were collected, then the proteins corresponding to the key genes were molecularly docked with the small molecular structures of clinically proven effective herbal tonics, and molecular dynamic simulations were performed to select the best candidates for pharmacological compounds.

**Results:**

Elevated ferritin levels in patients with GDM were verified using clinical data. The presence of ferroptosis in placental tissues of patients with GDM was confirmed using electron microscopy and western blotting. Ninety-nine key genes with the highest correlation with ferroptosis were identified from DEGs and weighted gene co-expression network analysis (WGCNA). Analysis using the Kyoto Encyclopedia of Genes and Genomes demonstrated that the DEGs were primarily involved in the oxidative phosphorylation pathway. The key genes were further screened by PPI; two key genes, SF3B14 and BABAM1, were identified by combining the gene corresponding to protein structure and function, followed by molecular docking and molecular dynamic simulation. *Coptis chinensis* was proposed as the best candidate for herbal treatment at the molecular level.

**Conclusion:**

This data revealed the presence of ferroptosis in patients with GDM and identified possible modulatory roles of ferroptosis-related genes involved in the molecular mechanisms of GDM, providing new insights into the pathogenesis of GDM, which also provided new directions for the systematic optimization of TCM formulas for the management and targeted treatment of GDM.

## 1 Introduction

Gestational diabetes mellitus (GDM) is traditionally defined as glucose intolerance of variable severity, with onset or first detection during pregnancy. The global prevalence of GDM is up to one in seven pregnancies (1). GDM is associated with long-term adverse health outcomes in women and their offspring and is at the center of the “diabetes begetting diabetes” vicious circle (2). For example, women with GDM experience an increased risk of hypertensive disorders in pregnancy, preterm delivery, and an exceptionally high risk for type 2 diabetes, metabolic syndromes, and cardiovascular diseases later in life (3). Children born to pregnant women affected by GDM often experience excess growth during the fetal period, resulting in increased adiposity at birth and an increased risk for obesity and cardiometabolic disorders later in life (2, 4). Current studies have demonstrated that the occurrence and development of GDM is influenced by multiple factors, among which genetic, metabolic and environmental factors are important in the pathogenesis of GDM(5). Currently, we recommend dietary modification and increasing appropriate physical activity to improve gestational diabetes, and when basic methods cannot control blood glucose, insulin therapy (6) or oral hypoglycemic agents, mainly metformin and glibenclamide (7), are mostly used. The majority of current studies have focused on hypoglycemic approaches to treat GDM. However, the efficacy in preventing and stopping further disease progression have not been completely satisfactory. Therefore, identifying women at increased risk of GDM at an early stage and exploring markers to predict GDM can aid in the diagnosis and treatment of GDM.

The average daily iron requirement during pregnancy increases to approximately 1,000–1,200 mg to meet the maternal and fetal placental needs for erythropoiesis, growth, and development (8). Therefore, much attention has been focused on the prevention of iron deficiency, that is anemia, and iron supplementation has become a routine recommendation for all women throughout pregnancy (9). Nonetheless, as early as 20 years ago, studies suggested that iron accumulation increases the risk of glucose intolerance in late pregnancy and iron overload may play a role in the development of GDM (10). Many subsequent clinical studies have demonstrated a correlation between disturbances in iron homeostasis during pregnancy and GDM. A recent meta-analysis included 32 prospective cohort or case-control studies investigating the relationship between serum iron metabolic markers and GDM, and higher levels of serum iron, ferritin, transferrin saturation, ferritin, and hemoglobin were reported in patients with GDM than in those without GDM, suggesting that high serum ferritin and hemoglobin levels are positively associated with the risk of developing GDM (11). Recent studies have questioned the recommendation of routine iron supplementation during pregnancy in iron-sufficient and non-anemic women and have further suggested that excessive iron intake may paradoxically increase the risk of reproductive disorders (12). Several relevant clinical, *in vitro*, and *in vivo* studies have validated the correlation between iron overload and the development of GDM (13, 14); nonetheless, the specific mechanisms by which iron overload causes GDM are not fully understood. Iron death is an iron-dependent process characterized by dysregulation of iron homeostasis, leading to excessive iron death, which is a recently described programmed cell death process mediated by iron-dependent lipid peroxidation of the cell membrane. Iron overload drives iron accumulation and the biological effects thereof (15). Iron overload during pregnancy leads to ferroptosis, which causes GDM (12). Therefore, iron overload may be presumed to play a crucial role in GDM. However, whether iron overload actually causes GDM or is simply a byproduct of iron death remains to be proven.

As a type of programmed cell death, ferroptosis is dependent on iron and induced by the accumulation of oxidatively damaged phospholipids. It is associated with the malfunction of glutathione-dependent antioxidant defenses mediated by glutathione peroxidase 4 (Gpx4) via different pathways (8, 10), resulting in an increased amount of reactive oxygen species (ROS). This in turn leads to a reduction in the metabolism of lipid peroxides catalyzed by Gpx4 and intracellular glutathione, which causes Fe2+ to oxidize lipids in a Fenton-like manner, resulting in the production of large amounts of ROS and promotion of ferroptosis (16). Recently, the importance of ferroptosis has been demonstrated in the antioxidant activity of pancreatic β-cells and insulin resistance (17). However, the specific role of ferroptosis in GDM currently remains elusive.

Traditional Chinese medicine (TCM) is an effective way to treat GDM with chemical diversity, effectiveness and few side effects (18). In recent years, TCM has made remarkable progress in the treatment of GDM, and a large number of effective formulas have been accumulated. However, due to the complexity of TCM components, the formulations of TCM vary greatly. Current TCM-related studies have focused on given formulations, herbs or single compounds, and we performed screening of specific core drugs for the treatment of GDM by transcriptomics, network analysis and computer virtualization.

In this study, clinical data were applied to reaffirm the positive association of ferritin with the development of GDM, confirming the occurrence of ferroptosis in the placental tissue of patients with GDM by using electron microscopy, and the expression of GPX4, SLC7A11, and FTH1 was evaluated by western blot analysis. Recent advances in sequencing technology have greatly facilitated genetic studies of GDM. Reliable differentially expressed genes (DEGs) were identified in GDM based on the GSE70493 dataset. A weighted gene co-expression network analysis (WGCNA) in 32 patients with GDM and 31 non-GDM controls was conducted. Compared with traditional methodologies that take every transcript in the microarray alone and only capture two less pieces of information than that provided by the microarray, WGCNA takes correlations among those transcripts into account and identifies potential disease-related gene co-expression modules (GCMs) by considering associations between GCMs and disease traits as well as intramodular associations. A gene module closely associated with ferroptosis was identified and screened for potential biomarkers via a combination of protein–protein interaction (PPI) networks. Wheat-flavored Rehmannia decoction, Astragalus Sijunzi decoction, Radix Astragali and Radix Ophiopogonis decoction, and Shenqi Dihuang decoction have been used for many years in the clinical treatment of GDM with remarkable efficacy. The complexity of Chinese herbal medicine suggests a potential for multiple therapeutic applications. We analyzed the herbs that could be evaluated for the treatment of GDM from the perspective of ferroptosis. In summary, we analyzed the impact of ferroptosis on GDM utilizing a bioinformatics approach and found a significant association between ferroptosis and GDM. In addition, we analyzed the herbs targeted ferroptosis to treat GDM based on molecular docking and molecular dynamic simulation. This study provided new directions for the management and targeted treatment of GDM.

## 2 Materials and methods

### 2.1 Collection of clinical data and tissue samples

**Figure 1:**
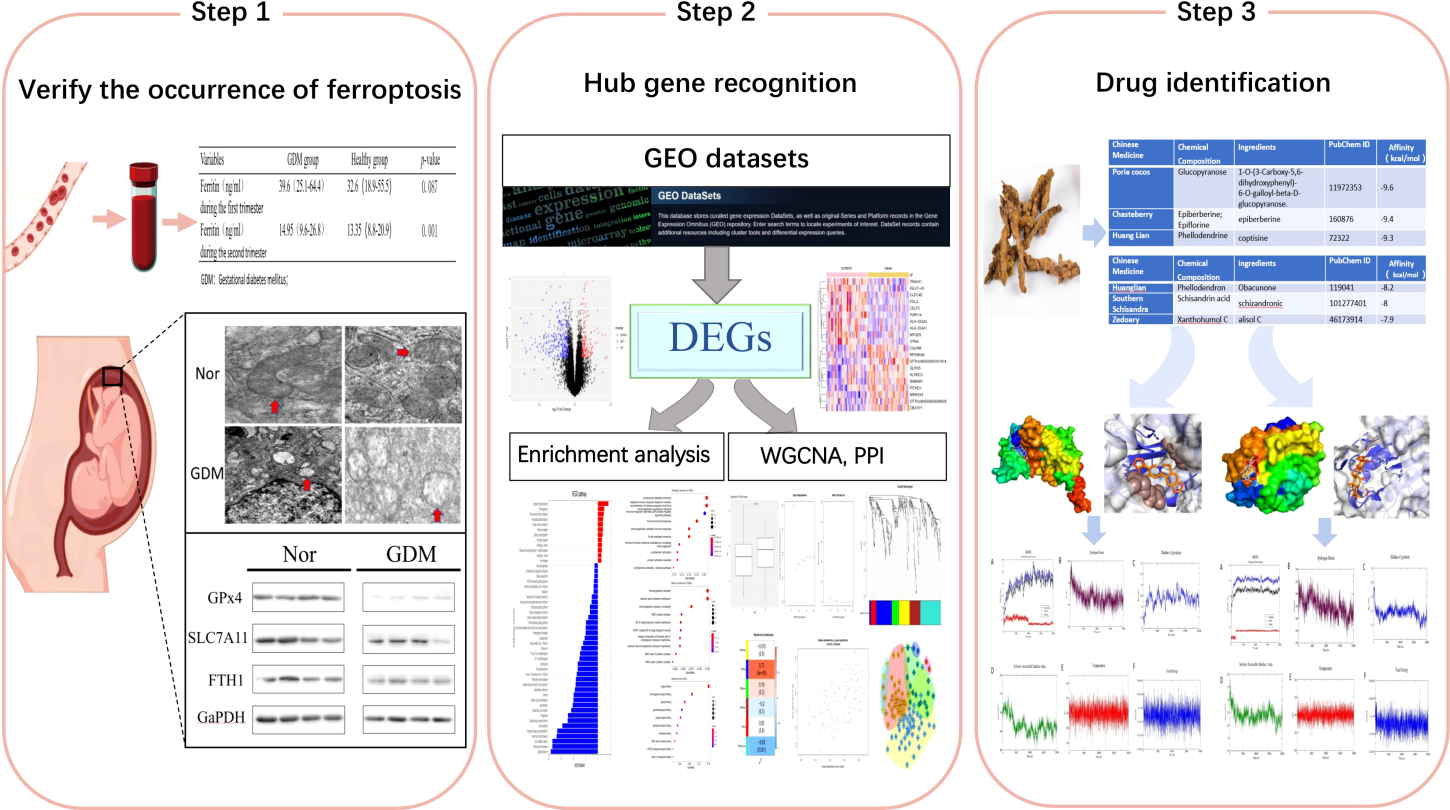
Flowchart.

A procedural flowchart of the study is illustrated in **Figure 1**. A retrospective analysis was performed from January 2020 to December 2020 at the Shengjing Hospital, China Medical University. Pregnant women who completed the oral glucose tolerance test (OGTT) from 24–28 weeks of pregnancy and received a diagnosis of GDM based on abnormal OGTT defined as per the criteria of the International Association of Diabetes and Pregnancy Study Groups (IADPSG) were included in the study; patients with normal OGTT in the same period were included as controls. A total of 65 GDM patients with ferritin and 153 normal pregnant women with ferritin were included during the first trimester. And 330 GDM patients with ferritin and 920 normal pregnant women with ferritin were included during the second trimester. Detailed information is listed in **Supplementary Table 1**. Subjects with a history of GDM, smoking before and during pregnancy, and medical conditions, such as hemoglobinopathies, infections, and other chronic diseases, were excluded. Serum ferritin levels were measured during early (8–12 weeks) and mid-pregnancy (24–28 weeks). Ferritin and blood glucose levels were measured using ferritin kit for the DXI800 instrument (Beckman Coulter, Brea, CA, USA) and blood glucose kit for the c16000 instrument (Abbott Diagnostics, Abbott Park, IL, USA).

Women with full-term singleton pregnancies who underwent cesarean delivery at the Shengjing Hospital of China Medical University, from January 2021 to June 2021, were selected. GDM was diagnosed based on the criteria of the IADPSG. The exclusion criteria included twin (multiple) pregnancies; diabetes diagnosis prior to pregnancy; pregnancy complications such as preeclampsia; essential (primary) hypertension; hyperthyroidism; hypothyroidism; Cushing’s syndrome; smoking; assisted reproduction; and severe liver, kidney, or heart disease. 10 women with GDM and 10 healthy controls were included in the study. Placental tissue samples from women with GDM and healthy women were collected randomly from the substrate (2 cm from the periphery to avoid placental infarction) immediately after delivery for western blotting. The tissue samples were washed repeatedly with phosphate buffered saline (PBS), placed in labeled lyophilized tubes, and stored in liquid nitrogen. Two or three pieces of placental tissue approximately 0.5 cm^3^ in size were randomly sampled from the substrate (in the middle of the central tufts of villi connecting to the maternal region) immediately after delivery for electron microscopic evaluation. The placental tissue samples were washed in saline, and the washed placental tissue was immersed in 2.5% glutaraldehyde fixative (0.1 mol/L sodium dimethylarsonate buffer, pH 7.4). The tissue blocks were cut into 1 mm^3^ pieces and fixed at 4°C for at least 2 h. Sampling was performed simultaneously by YW and BL. This study was conducted in accordance with the principles of the Declaration of Helsinki and approved by the Research Medical Ethics Committee of Shengjing Hospital of China Medical University (The ethics number of the experiment:2017PS066K).

### 2.2 Validation of ferroptosis in placental tissues of patients with GDM

Electron microscopy was used to examine mitochondrial changes in placental tissues of patients with GDM. After fixation, the placental tissues were washed with 0.1 mol/L PBS for 30 min and post-fixed with 1% osmium tetroxide fixative for 90 min at 4°C. After rinsing, dehydration, permeabilization, embedding, sectioning, and staining, changes in mitochondrial morphology in the placental tissue cells were observed under an electron microscope (H-7650, Hitachi, Japan) and photographed for sample retention. Protein levels associated with ferroptosis were subsequently evaluated. Placental tissues collected from patients with GDM (n = 10) as well as from normal pregnancies (n = 10) were removed from the liquid nitrogen, and the samples were lysed with RIPA lysis buffer (P0013B, Beyotime, Beijing, China) and phenylmethylsulfonyl fluoride (PMSF). Proteins in the lysates were electrophoresed on 10% sodium dodecyl sulfate-polyacrylamide gel electrophoresis (SDS-PAGE) and subsequently transferred to polyvinylidene fluoride (PVDF) membranes. After closure with 5% skim milk to block non-specific binding, the specific primary antibody was placed and incubated overnight at 4°C, followed by incubation at 37°C for 1 h with horseradish peroxidase-labeled secondary antibodies (Cell Signaling Technology). The following primary antibodies were used: SLC7A11 (1:500; ab175186; Abcam), GPX4 (1:1000; ab231174; Abcam), FTH1 (1:500; ab75972; Abcam), and GAPDH (60004-1-Ig; Proteintech).

Antibody-labeled proteins were detected using chemiluminescence with a LAS-3000 luminescence image analyzer (Fujifilm Holdings Corporation, Tokyo, Japan) and SuperSignal West Femto maximum sensitivity substrate (Thermo Fisher Scientific, Inc., Waltham, MA, USA). Using the gel image processing software image J, the backgrounds were removed, units and assay data were adjusted, and grayscale values were calculated. Values were entered into GraphPad Prism 7 (GraphPad Software 7.0.1, San Diego, CA, USA) for statistical analyses. Experiments were performed in triplicate for each target protein strip. Antibody-labeled proteins were detected using chemiluminescence with a LAS-3000 image analyzer (Fujifilm Holdings Corporation, Tokyo, Japan), and ImageJ was used to remove the background, adjust the units with the assay data, and calculate the grayscale values. The values were entered into GraphPad Prism 7 for statistical analysis. Triplicate experiments were performed for each target protein strip.

### 2.3 Microarray data source

GSE70493 with the GPL17586 platform was collected from Gene Expression Omnibus (GEO) (https://www.ncbi.nlm.nih.gov/geo/). In total, 63 samples were obtained, including RNA-seq data and clinical data; 32 placenta samples from patients with GDM and 31 placenta samples from patients without GDM, and 253 ferroptosis-related regulators were collected from the FerrDb database (19).

### 2.4 Identification of differentially expressed genes

Following potent mean background correction, the microarray matrix file was subjected to quantile normalization and expression calculation using Affymetrix to obtain gene expression profiles. The differentially expressed genes (DEGs) between placental samples from 32 patients with GDM and 31 matched pregnancies without GDM were identified using the R package Limma (available online: http://www.bioconductor.org/). The criteria for DEGs considered included |log2(FC)| > 0.1 and a *p*-value < 0.05. Subsequently, a heatmap of the obtained differential genes was constructed using the pheatmap package (version 1.0.8, https://cran.r-project.org/web/packages/pheatmap).

### 2.5 Functional enrichment analysis

The Kyoto Encyclopedia of Genes and Genomes (KEGG) pathway and Gene Ontology (GO) analyses were used to investigate underlying potential mechanisms; the GO analysis consisted of biological process (BP), cellular component (CC), and molecular function (MF). This analysis was performed using the R package ClusterProfiler. In this study, GO and KEGG pathway enrichment analyses were performed for the DEGs obtained. False discovery rate (FDR) <0.05 was identified as the threshold for enrichment analysis.

### 2.6 Weighted gene co-expression network analysis

A weighted gene co-expression network was constructed to explore the correlation with ferroptosis. The co-expression similarity network was composed of the GSE70493 mRNA expression profile and 253 ferroptosis-related regulators, using the R package WGCNA. The soft thresholding was set at seven. A matrix of weighted adjacency was created using the formula amn = |cmn|β (amn: adjacency between gene m and gene n; cmn: Pearson’s correlation; β: soft-power threshold). Subsequently, the clinical trait data were loaded, and scale independence and mean connectivity were estimated. Additionally, the topological overlap measure (TOM) matrix, transformed by the adjacency matrix, was used to estimate the connectivity property in the network. A hierarchical clustering dendrogram of the TOM matrix was constructed using the average distance with a minimum size threshold of 30 to classify similar gene expression profiles into different gene modules. Different module eigengenes (MEs) and clinical traits were then correlated. The gene significance (GS) quantifying the associations of individual genes with the clinically interesting trait and module membership (MM), which acted as the correlation between the MEs and the gene expression profiles, were calculated. As previously reported, if GS and MM were highly correlated, the most important (central) elements in the modules were also tightly associated with the trait. As such, they can be used to construct the PPI network with the use of Cytoscape software to identify hub genes.

### 2.7 Molecular docking and molecular dynamics analysis

To evaluate whether Wheat-flavored Rehmannia decoction, Astragalus Sijunzi decoction, Radix Astragali and Radix Ophiopogonis decoction, and Shenqi Dihuang decoction tonics can bind to the ferroptosis-related genes and play a therapeutic role, molecular docking of key active ingredients with key targets was performed, and the binding energies were evaluated as evaluation indices. Given the complexity of the active ingredients of herbal medicines, we screened all active ingredients based on multiple commonly used herbal ingredients (https://old.tcmsp-e.com/index.php; http://www.tcmip.cn/TCMIP/index.php/Home/; http://119.3.41.228:8000/tcmid/herb/1138/).The molecular structures of the akey active ingredients of all Chinese medicine components involved in the four tonics were downloaded from the PubChem database (https://pubchem.ncbi.nlm.nih.gov/), along with a total of 218 ingredients. After format conversion using Open Babel, AutoDockTools 1.5.7 was used to process the ligands and receptors. Core target protein structures were downloaded from the protein database (PDB; http://www.rcsb.org/pdb/home/home.do). We used PyMol software to remove water molecules and residues from the receptor proteins, followed by the addition of nonpolar hydrogens to generate coordinate files using AutoDockTools 1.5.7. Protein-binding pockets were predicted using DoGSiteScorer (https://proteins.plus/). Using the key target as the receptor and the corresponding key active ingredient as the ligand, AutoDockTools was used to set the docking box center and the box grid parameters, including the active pocket sites where the small molecule ligand might bind. Molecular docking was performed using AutoDock Vina 1.1.2, maintaining the top 10 ligands with the lowest binding free energy. The lowest energy conformation was selected as the best binding conformation, and the protein-ligand forces were mapped in two dimensions using Poseview. The binding sites, interaction forces between amino acid residues, and binding pocket surfaces were analyzed in three dimensions using PyMOL. The molecular structures of 218 active ingredients were obtained by docking, and the best-scoring-complex conformation was applied for mapping using PyMOL.

Based on the results of molecular docking, molecular dynamic simulations of the obtained protein-ligand complexes were performed using GROMACS 5.0 software (http://www.gromacs.org/); GROMOS was used to generate input files, where the topology files of the small-molecule ligands were generated using the ATB website (http://atb.uq.edu.au/index.py). All simulations were performed using periodic boundaries, and all were placed in a dodecahedral cell box (with a 1 nm edge distance). The simple point charge solvent model was initially added as a water model, and five chloride ions were added to the solventized system to maintain the electrical neutrality. The simulations were performed using a periodic boundary approach with 1,000 steps of energy minimization at 300 K for the protein and small-molecule ligand complexes, followed by 100 ps canonical ensemble (NVT) systemic equilibrium and 100 ps of isothermal–isobaric (NPT) systemic equilibrium for the optimized system, in which the system positions were constrained. Finally, the entire system was maintained at 300 K for 20 ns of kinetic simulations, where the time interval was 2 fs, and the coordinate trace files were saved every 10 ps. The post-simulation analysis included structure comparison (alignment), calculation of root mean square deviation (RMSD), hydrogen bonding (H-bond), solvent accessible surface area (SASA), temperature, total energy, and radius of gyration (Rg). The RMSD was used as an evaluation index of the stability of the complexes; the smaller the average RMSD, the more stable the bonding.

### 2.8 Statistical analysis

All analyses were performed using R software (version 3.6.1, www.r-project.org). All statistical tests were two-sided, and statistical significance was set at *p* < 0.05. Group comparisons were performed using the independent t-test for normally distributed variables and the Mann–Whitney U test for variables showing an abnormal distribution. The statistical methods and algorithms used were described in the corresponding steps.

## 3 Results

### 3.1 Validation of ferroptosis in patients with GDM

**Figure 2:**
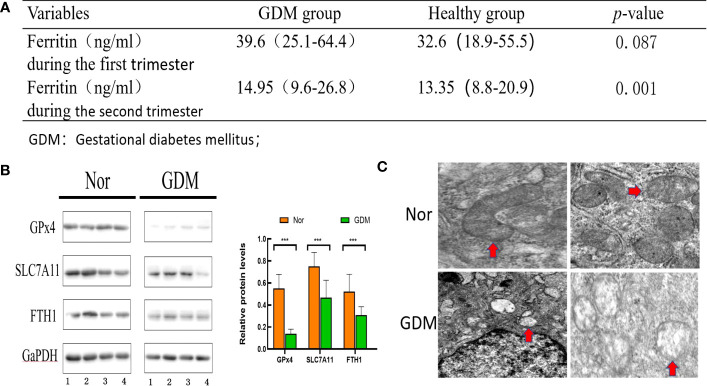
Evaluation of ferroptosis in GDM patients. **(A)** ferritin in early and mid-pregnancy between the GDM group and non-GDM group. **(B)** Levels of proteins associated with ferroptosis were assessed using western blot between the GDM group (n = 10) and non-GDM group (n = 10). **(C)** Mitochondrial morphology was observed under electron microscopy between the GDM group and non-GDM group(Red arrows point to changes in mitochondria. Mitochondria in GDM placental tissue conform to ferroptosis changes, with reduced or disappeared mitochondrial cristae and rupture of mitochondrial outer membrane; Mitochondria in normal placental tissue are normal). ***p<0.001.

Retrospective analysis of ferritin levels measured in early and mid-pregnancy between the GDM and healthy groups (**Figure 2A**) demonstrated that serum ferritin levels in early pregnancy were positively associated with the risk of GDM and serum ferritin levels in mid-pregnancy were significantly higher in pregnant women with GDM than in non-GDM women (*p* < 0.05). Thus, our results are consistent with the findings of previous studies indicating that high ferritin levels are an important factor in GDM development. To further investigate whether ferroptosis was activated during GDM, western blotting to detect the levels of protein associated with ferroptosis and electron microscopic observation of changes in intracellular mitochondria were performed. As depicted in **Figure 2B**, western blotting indicated reduced levels of GPx4 (p < 0.01), SLC7A11 (p < 0.01), and FPN1 (p < 0.01) in placental tissues of patients with GDM compared with healthy controls. Mitochondrial morphology was observed using electron microscopy: mitochondria were swollen, and mitochondrial cristae were reduced or absent (**Figure 2C**). Overall, this evidence suggests that iron death is activated during GDM.

### 3.2 Identification of DEGs in GDM

**Figure 3:**
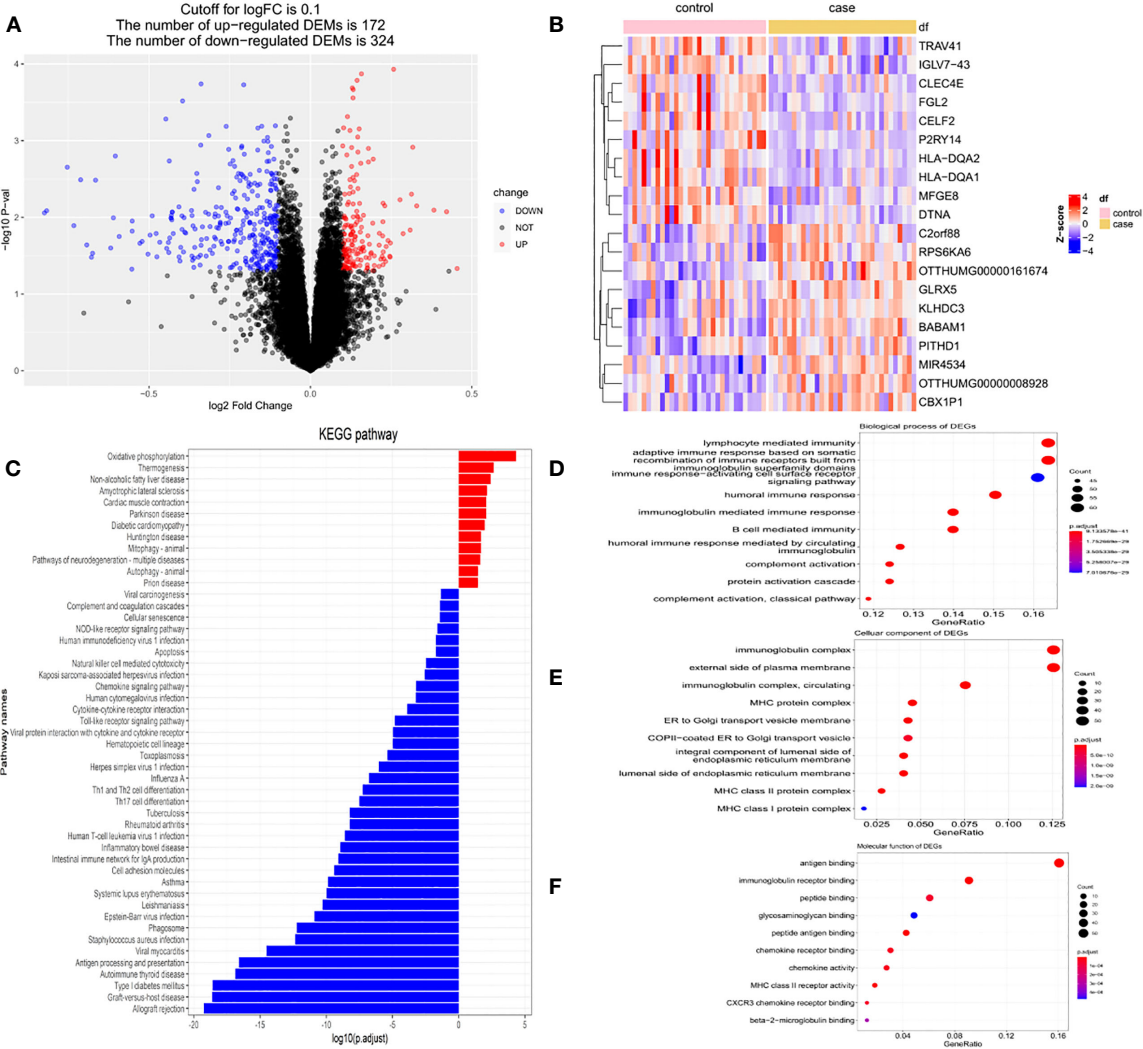
The DEGs based on GEO mRNA microarray datasets (GSE70493). **(A)** The volcano plot of DEGs in GSE70493. **(B)**The heatmap of top 200 DEGs in GSE70493. **(C)** KEGG enrichment analysis. **(D)** GO enrichment analysis of biological process. **(E)** GO enrichment analysis of cellular component. **(F)** GO enrichment analysis of molecular function.

In this study, DEGs and their significant biological characteristics were identified based on GEO mRNA microarray datasets (GSE70493); there were 32 GDM cases and 31 matched pregnancies, without maternal complications. Following gene expression assays, data processing, and normalization, DEGs were screened among each mRNA dataset using the Limma R package with the criteria of |log2(FC)| > 0.1 and a *p*-value <0.05. The volcano plot contained each of the DEGs in the dataset, and 172 upregulated and 324 downregulated genes were identified (**Figure 3A**). The DEGs are listed in **Supplementary Table 2**.

A heatmap was compiled for the top 20 genes with the most significant differential expression from the 63 samples. Cluster analysis suggested that these DEG expression profiles were able to distinctly distinguish GDM from non-GDM samples (**Figure 3B**).

Subsequently, functional enrichment analysis was performed to identify the signaling pathways differentially affected by GDM using the ClusterProfiler package. The DEGs between the GDM group and the normal group were chiefly enriched in “oxidative phosphorylation” pathways, “thermogenesis” pathways, and “non-alcoholic fatty liver” disease pathways as determined using KEGG analysis, while the group without GDM was highly associated with “allograft rejection” pathways, and “graft-versus-host disease” pathways (**Figure 3C**). The GO analysis of the DEGs demonstrated that the differential genes were primarily enriched in the “lymphocyte-mediated immune” pathway, “adaptive immune response based on somatic” pathway, and “recombination of immune receptors built from immunoglobulin superfamily domains” pathway in BP (**Figure 3D**). In terms of cellular components, the DEGs were primarily enriched in the immunoglobulin complex, external side of plasma membrane, and circulating immunoglobulin complex (**Figure 3E**); with respect to MF, the DEGs were primarily enriched in antigen binding, immunoglobulin receptor binding, and peptide binding (**Figure 3F**).

### 3.3 Genes co-expressed with ferroptosis-related genes via WGCNA

**Figure 4:**
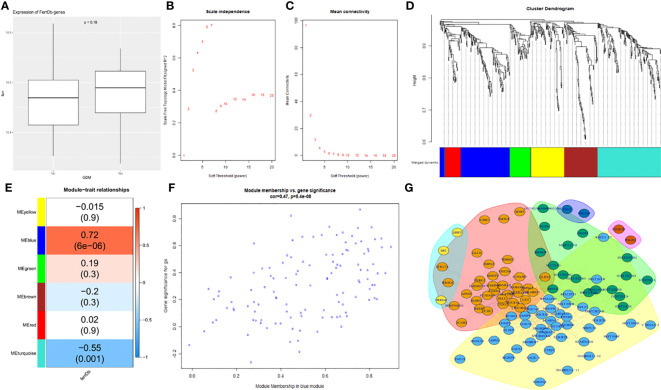
Screening of key ferroptosis-related genes using WGCNA and PPI network. **(A)** The differences in expression of ferroptosis signature genes between the two groups. **(B)** The correlation between various soft-thresholding power and scale-free topology model fit index. **(C)** The correlation between various soft-thresholding power and mean connectivity (degree). **(D)**The dendrogram clustered using topological overlap based on ferroptosis signature genes and mean mRNA expression of DEGs. **(E)** The correlation among different modules and traits. **(F)** The analysis of bule module genes and GDM. **(G)** PPI network.

To determine whether ferroptosis affects GDM, 253 ferroptosis-related regulators from FerrDb were collected and a co-expression network based on the ferroptosis signature genes and average expression values of 496 differential genes was constructed (**Supplementary Table 3**). Differences in the expression of ferroptosis signature genes between the two groups was compared. The ferroptosis signature expression was greater in the GDM group than in the non-GDM group (**Figure 4A**). Then, using 10 as a soft threshold (**Figures 4B, C**), a co-expression network with six modules using DEGs in the GDM group was constructed with the WGCNA package (**Figure 4D**). The clustering results between modules demonstrated that the blue module, which contained 99 genes, showed the highest correlation with ferroptosis-related genes (**Figure 4E**; r = 0.72, P = 6e-06), and genes from this module were selected as ferroptosis co-expression genes.

The correlation between DEGs and the members in the blue module, as well as that between the genes and ferroptosis was analyzed individually; additionally, the two variables were plotted as abscissa and ordinate, and the result was positively correlated. The blue modules were again highly correlated with ferroptosis (**Figure 4F**).

A PPI network was then constructed to explore the hub genes (**Figure 4G**). Notably, COX7A2, NDUFA6, UQCR10, UXT, SELT, SF3B14, BABAM1, SUPT4H1, and SCARNA12 were revealed as the top 10 hub genes according to the decreasing order of “Vlength” (illustrated in **Supplementary Table 4**).

### 3.4 Identification of potential ferroptosis-related hub gene drug targets

Based on the top 10 hub genes obtained, the proteins corresponding to SF3B14 and BABAM1 were determined to be potential drug targets in terms of protein structure and function. The core target protein structures were downloaded from the PBD protein database (http://www.rcsb.org/pdb/home/home.do). Based on the source of the structure, complexity of the structure, influencing factorsand, We finally chose BABAM1_6H3C and SF3B14_2F9D. Two protein structures were docked with each of the 218 Chinese herbal ingredients in the four tonics. AutoDock evaluated the energy match between the receptor and ligand based on a semi-empirical free energy calculation method; the smaller the value, the greater the affinity between the ligand and receptor (20). Molecular docking of protein SF3B14_2F9D with 218 herbal components and the top 10 best results for binding free energy are listed in **Table 1**. The top 10 binding free energy produced negative results for 218 docking experiments, and the binding energy of the best top 3 docking results were -9.6, -9.4, and -9.3 kcal/mol, indicating that protein SF3B14_2F9D had good binding effects with glucopyranoside, epifriedrine, and pyrantel bases. Molecular docking of BABAM1_6H3C with 218 herbal components was then performed, and the top 10 best results for the binding free energy are listed in **Table 2**. The top 10 binding free energy results were negative among the 218 docking experimental results, and the binding energy of the best top 3 docking results were -8.2, -8, and -7.9 kcal/mol, indicating that protein BABAM1 had good binding effect with *Coptis chinensis*, Nanwuji, and Zedoary. Combining drug efficacy and drug binding efficacy, we determined that *Coptis Chinensis* demonstrated the optimal binding energy.

**Table 1:** The TOP10 docking binding energy between 218 herbal components and the active site of the protein target (SF3B14_2F9D).

Chinese Medicine	Chemical Composition	Ingredients	PubChem ID	Affinity (kcal/mol)
Poria cocos	Glucopyranose	1-O-(3-Carboxy-5,6-dihydroxyphenyl)- 6-O-galloyl-beta-D-glucopyranose.	11972353	-9.6
Chasteberry	Epiberberine; Epiflorine	epiberberine	160876	-9.4
Huang Lian	Phellodendrine	coptisine	72322	-9.3
Radix Codonopsis	Chrysoprase	Chrysanthemaxanthin	21160900	-9.3
Fructus Schisandrae	Neonanwort B	neokadsuranic acid B	145709631	-9.2
Yam	Phellodendrin	Doradexanthin	16061189	-9.1
Huanglian	Pawpaw rhubarb dianthrone	Palmidin A	5320384	-9
Cornus officinalis	N	galloyl(-3)[galloyl(-6)]a-All	6398541	-9
Yellow Essence	Diosgenin	diosgenin	99474	-9
Coptis Chinensis	Phellodendron	Obacunone	119041	-9

N, none.

**Table 2:** The TOP10 docking binding energy between 218 herbal components and the active site of the protein target (BABAM1_6H3C).

Chinese Medicine	Chemical Composition	ingredients	PubChem ID	Affinity (kcal/mol)
Coptis Chinensis	Phellodendron	Obacunone	119041	-8.2
Southern Schisandra	Schisandrin acid	schizandronic	101277401	-8
Zedoary	Xanthohumol C	alisol C	46173914	-7.9
Huanglian	Phellodendrine; Berberine	coptisine	72322	-7.8
Southern Schisandra	Neo-South schisanic acid	neokadsuranic acid B	145709631	-7.8
Mai Dong	Hydroxymethyl maidenhair flavonoid A	Hydroxymethylophiopogonone A	101238141	-7.7
Mai Dong	Macroisoflavone A	Ophiopogonone A	10087732	-7.5
Yam	Schisandrin; Dihydroquercetin; Flavopiridol; Yewaxanthin;	taxifolin	712316	-7.5
Mai Dong	Maidenhair Flavonoid A	Ophiopogonanone A	92449512	-7.5
Chasteberry	Pinusolitrin; Dihydroquercetin; Flavopiridol; Yewcitrin;	taxifolin	439533	-7.4

### 3.5 Molecular docking and molecular dynamics simulation of targeted drugs

**Figure 5:**
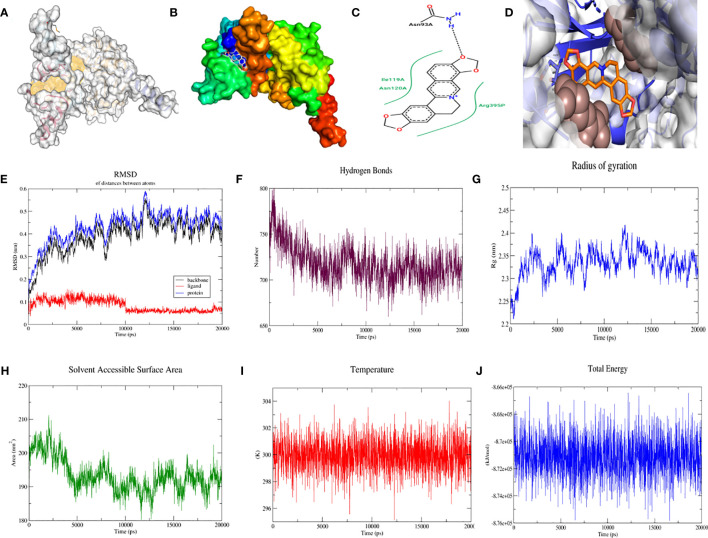
Molecular docking and molecular dynamics simulation of SF3B14_2F9D with coptisine. **(A)** Location of the predicted binding pocket of SF3B14_2F9D; **(B)** Surface map of the docking result of SF3B14_2F9D with coptisine; **(C)** Two-dimensional structure of the docking result of SF3B14_2F9D with coptisine; **(D)** Three-dimensional structure of the docking result of SF3B14_2F9D with coptisine; **(E)** RMSD between SF3B14_2F9D and coptisine using molecular dynamics simulation; **(F)** Hydrogen bonding network between SF3B14_2F9D and coptisine using molecular dynamics simulation **(G)** Gyration radius between SF3B14_2F9D and coptisine using molecular dynamics simulation; **(H)** Solvent accessible surface area between SF3B14_2F9D and coptisine using molecular dynamics simulation; **(I)** Temperature between SF3B14_2F9D and coptisine using molecular dynamics simulation; **(J)** Total energy between SF3B14_2F9D and coptisine using molecular dynamics simulation;.

We further validated the stability and biological activity of SF3B14_2F9D with coptisine, a component of *Coptis Chinensis*, BABAM1_6H3C, and berberine, a component of *Coptis Chinensis*. Initially, SF3B14_2F9D was docked with coptisine to form more hydrophobic interactions and predict the location of the protein binding pocket as depicted in **Figure 5A**, followed by the establishment of the binding surface map (**Figure 5B**), two-dimensional spatial model (**Figure 5C**) and three-dimensional spatial model (**Figure 5D**). As illustrated in the figure, coptisine bound to SF3B14_2F9D in the predicted binding pocket. The molecular dynamics of SF3B14_2F9D with coptisine were then further simulated. In the 20 ns molecular dynamics simulation, the protein SF3B14_2F9D reached the equilibrium state at 0.45 nm, and coptisine reached the equilibrium state (tuA) at 0.075 nm based on RMSD over time (**Figure 5E**). Molecular dynamic simulations of the equilibrium trajectories were used for further analysis; SF3B14_2F9D and coptisine were able to form 725 hydrogen bonds (**Figure 5F**). Combining the radius of gyration (**Figure 5G**), SASA (**Figure 5H**), temperature (**Figure 5I**), and total energy (**Figure 5J**) between SF3B14_2F9D and coptisine, the results suggest that SF3B14_2F9D can stably bind to coptisine and exert potential biological activity.

**Figure 6:**
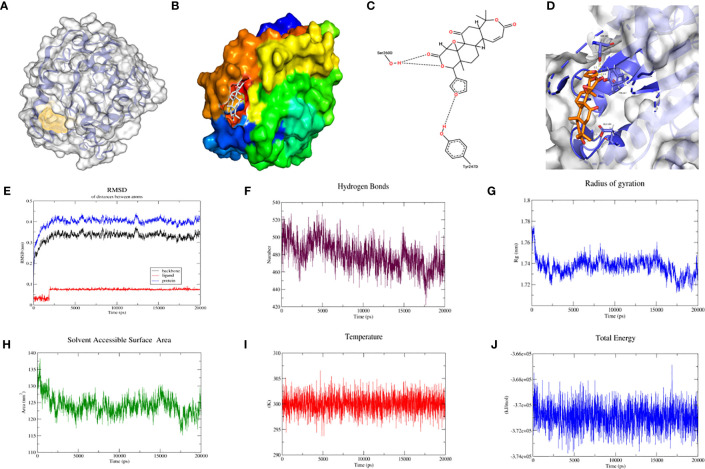
Molecular docking and molecular dynamics simulation of BABAM1_6H3C with berberine. **(A)** Location of the predicted binding pocket of BABAM1_6H3C; **(B)** Surface map of the docking result of BABAM1_6H3C with berberine; **(C)** Two-dimensional structure of the docking result of BABAM1_6H3C with berberine; **(D)** Three-dimensional structure of the docking result of BABAM1_6H3C with berberine; **(E)** RMSD between BABAM1_6H3C and berberine using molecular dynamics simulation; **(F)** Hydrogen bonding network between BABAM1_6H3C and berberine using molecular dynamics simulation **(G)** Gyration radius between BABAM1_6H3C and berberine using molecular dynamics simulation; **(H)** Solvent accessible surface area between BABAM1_6H3C and berberine using molecular dynamics simulation; **(I)** Temperature between BABAM1_6H3C and berberine using molecular dynamics simulation; **(J)** Total energy between BABAM1_6H3C and berberine using molecular dynamics simulation;.

As with the process above, protein BABAM1_6H3C was again docked with berberine to form more hydrophobic interactions, predict the location of the protein binding pocket as illustrated in **Figure 6A**, and establish the binding surface map (**Figure 6B**), two-dimensional spatial model (**Figure 6C**) and three-dimensional spatial model (**Figure 6D**). As depicted in the figure, berberine was able to bind to BABAM1_6H3C in the predicted binding pocket. Subsequently, molecular dynamic simulations of BABAM1_6H3C with berberine were performed. In the 20 ns molecular dynamic simulation, protein BABAM1 reached equilibrium at 0.4 nm, and berberine reached equilibrium at 0.075 nm based on RMSD over time (**Figure 6E**). Subsequently, BABAM1_6H3C and berberine formed 725 hydrogen bonds (**Figure 6F**). Combining the radius of gyration (**Figure 6G**), solvent-accessible surface area (**Figure 6H**), temperature (**Figure 6I**), and total energy (**Figure 6J**), the results suggest that BABAM1_6H3C can be stably bound to berberine and exert potential biological activity.

## 4 Discussion

One of the most common complications of pregnancy worldwide is GDM, and it causes many long-term maternal and neonatal complications affecting 2%–22% of all pregnancies (21). Adverse consequences of GDM for both mother and fetus can lead to damage or functional disorders in various organs of the pregnant woman and are associated with adverse fetal outcomes such as macrosomia, pre-eclampsia, obstructed shoulder birth, stillbirth, neonatal hyperbilirubinemia, neonatal hypoglycemia, and respiratory distress (22, 23). Patients with GDM and newborns with GDM are at increased risk of obesity, type 2 diabetes, and other metabolic diseases in the long term (24). Despite tremendous progress in its treatment, GDM still has adverse effects on the long-term health of both the mother and her offspring. The pathogenesis of GDM is influenced by multiple factors, among which epigenetic alterations are important mechanisms affecting the development of GDM. The placenta serves as an important connecting organ that maintains normal fetal growth and development, providing nutrition, oxygen, and other essential factors to the fetus. The placenta maintains a normal pregnancy by selectively regulating and transporting nutrients and vital ions. A common complication of pregnancy is GDM and various studies have demonstrated a correlation between impaired iron homeostasis and GDM (13, 25). Studies have reported that ferroptosis may play a central role in major placenta-related obstetric disorders (26). The results of a meta-analysis that included 10 studies showed that the risk of GDM increased with increasing ferritin (27). Higher levels of ferritin in mid-trimester are significantly associated with risk of GDM and adverse pregnancy outcomes associated with GDM (28). Ferroptosis has been reported to impair islet cell viability and function; ferroptosis inhibitors can reverse this damage (29). Hyperglycemia reportedly leads to impaired iron transport and increased lipid peroxidation. However, treatment with the antioxidant sodium selenite (NaSe) altered the expression of iron homeostasis genes, suggesting that hyperglycemia is involved in the induction of ferroptosis (14). These features suggest that ferroptosis is closely related to the development of GDM. We hypothesized that dysregulation of iron homeostasis genes in placental tissues and ferroptosis are likely potential mechanisms for the development of GDM. The aim of this study was to determine the correlation between GDM and ferroptosis through clinical studies, molecular biological data, and bioinformatics analysis, with the aim of identifying targets for the early treatment of GDM.

In this study, the correlation between GDM and serum ferritin levels was investigated, and increased iron levels inducing the development of ferroptosis in placental tissue and alteration of the protein expression of ferroptosis-related genes were subsequently verified. The results of the clinical correlation analysis demonstrated that serum ferritin levels were higher in patients with GDM than in patients without GDM in both early and mid-pregnancy, and there was a significant difference in mid-pregnancy serum ferritin levels between the two groups. Ferroptosis is characterized by the onset of mitochondrial dysfunction, primarily in the form of mitochondrial swelling, with a reduction in cristae and increased mitochondrial membrane permeability (30). We observed the mitochondrial ultrastructural changes in trophoblasts in the GDM and normal groups using electron microscopy. The mitochondria within trophoblast cells appeared significantly swollen and were accompanied by specific changes in the disappearance or reduction of cristae. Changes in the expression of ferroptosis-related genes in placental tissues were subsequently measured. Western blotting demonstrated reduced expression of GPx4, SLC7A11, and FTH1 in placental tissues of patients in the GDM group. These results confirm the presence of elevated ferritin levels and occurrence of ferroptosis in placental tissues in patients with GDM. The alteration of expression of ferroptosis-related genes in placental tissues was then verified.

DEGs are generally recognized as an important feature of disease progression. The genes involved in the pathogenesis of GDM and associated with ferroptosis were examined; 172 upregulated and 324 downregulated genes were identified by analyzing the differential expression profiles of placental tissues from GDM and non-GDM samples. Clustering analysis was then performed; it was observed that these DEG profiles clearly distinguished between GDM and non-GDM samples. The differential expression of these genes provides a new direction for further exploration of the underlying molecular mechanisms involved in GDM. To reveal the potential functions of the DEGs, functional enrichment analysis was performed using GO analysis by database for annotation, visualization and integrated discovery and KEGG pathway analysis by gene set enrichment analysis. The functions of DEGs in three components, CC, MF, and BPs, were revealed using GO analysis. Following annotation of the genes themselves, the pathways involved in DEGs were explored using KEGG analysis; this revealed that the “oxidative phosphorylation” pathway was significantly activated in the GDM group. Under aerobic conditions, cells obtain energy mainly through oxidative phosphorylation, and during hypoxia, they undergo glycolysis (31). Mitochondria are an important source of ROS in human cells, and glycolysis is increased when mitochondrial metabolism is reduced. Increased mitochondrial ROS production reportedly promotes ferroptosis, and the application of mitochondria-targeted antioxidants or enzyme inhibitors can inhibit this process (32). The tricarboxylic acid cycle is an enzymatic pathway located in the mitochondrial matrix that transfers electrons to the mitochondrial electron transport chain through a series of redox reactions, resulting in the production of ATP through oxidative phosphorylation. Altered ATP/ADP ratios play a dual role in ferroptosis (32). Deficient oxidative phosphorylation may lead to iron sagging. These findings suggest that the “oxidative phosphorylation” pathway, which is significantly activated by differential genes, is closely related to the occurrence of ferroptosis in GDM. Placental development requires a unique oxidative stress environment, and when an imbalance of oxidative stress occurs in the placenta, lipid metabolism in placental tissues is disrupted, which can promote the onset of ferroptosis. Within the last several years, studies have described the interrelationship between maternal iron status, placental lipid metabolism, hyperglycemia and oxidative stress, which may be attributed to the effects or outcomes of ferroptosis. It has been found that synergistic mechanism of obesity before and during pregnancy increases inflammation and lipid peroxidation in trophoblasts which triggers a ferroptotic impairment of pancreatic islet function, resulting in GDM. High glucose conditions, reduced glutathione levels, impaired iron transport, and increased lipid peroxidation in trophoblast cells suggest that hyperglycemia causes ferroptosis. Iron accumulation may lead to increased lipid peroxidation and intracellularly generated protein carbonation, which in turn causes ferroptosis. The synergistic relationship between hyperglycemia and altered placental iron homeostasis during pregnancy was suggested as the mechanistic basis for ferroptosis (33). Although several clinical studies have reported a correlation between GDM and elevated maternal iron plasma levels, however, the associated pathophysiologic links and their underlying mechanisms are unclear. The fact that current studies on ferroptosis macrosomia have only scratched the surface of the phenomenon and its outcome remains a challenge for precision medicine, with detailed roles for iron prolapse in the onset and progression of GDM needing in-depth study.

Gene association patterns described by WGCNA explore the relationship between phenotypic data and gene modules based on the endogeneity of gene sets and identify the hub genes in the modules. The PPI network was then used to filter out genes that played a key role in this process. The expression of ferroptosis genes in both groups was analyzed, and the results confirmed the ferroptosis signature expression was greater in the GDM group than in the non-GDM group, and the changes were not statistically significant. Subsequently, WGCNA was applied to rapidly identify GCMs with the highest correlation with ferroptosis characteristics. The clustering results of the six modules constructed demonstrated that the blue module containing 99 genes was included in the PPI network to determine the pivotal genes due to the highest correlation with ferroptosis-related genes. Finally, 10 hub genes were identified. Based on the top 10 hub genes obtained, the use of the proteins corresponding to SF3B14 and BABAM1 as drug targets in terms of protein structure and function was determined. Homo sapiens splicing factor 3b subunit 14(SF3B14), also named SF3B6,encodes a 14 kDa protein subunit of the splicing factor 3b complex. This protein interacts directly with the adenosine that carries out the first transesterification step of splicing at the pre-mRNA branch site. It is well known that splicing of pre-mRNA is a critical step in gene expression and is accomplished by the spliceosome. The spliceosome specifically recognizes pre-mRNA, where U1 snRNP recognizes 5′-SS, U2 snRNP recognizes intronic Branch Site (BS). SF3B6 is involved in mRNA processing and RNA metabolism(34)。Sf3b6 stabilizes the BS:U2 snRNA duplex, contributing to the recognition of intronic (35). In addition, it has been found that functional knockdown of SF3B14 results in normal mulberry development but failure to form a blastocyst cavity or morphologically differentiated trophoblast ectoderm, which correlates with subsequent placentation (36). Previous studies have found that LUC7L2 is involved in coding for snRNP to affect pre-mRNA splicing and gene expression. The largest differential gene in the splicing analysis after knockdown of LUC7L2 is SLC7A11, which is required for SLC7A11 (xCT) splicing. LUC7L2 deletion leads to a reduction in SLC7A11 (xCT) and controls glycogen and glutamate metabolism (37), and promotes ferroptosis (25). It is reasonable to speculate that SF3B14, which is involved in pre-mRNA splicing, may contribute to ferroptosis by affecting the expression of key proteins for ferroptosis, which deserves to be further investigated in the future. BRISC and BRCA1 A complex member 1(BABAM1), locates in cytosol and nuclear body and involved in several processes, including mitotic G2 DNA damage checkpoint signaling; protein K63-linked deubiquitination; and positive regulation of DNA repair. Previous studies found that BABAM1 is regulated by mTORC2 to initiate DNA damage response, which can lead to ferroptosis (38). In addition, the kinase complex mTORC2 was found to prevent ferroptosis (39). Further studies can be conducted in the future both *in vivo* and *in vitro*.

We further verified the differential expression of SF3B14 and BABAM1 in women with GDM and normal pregnancy based on the GSE70493 dataset. SF3B14 and BABAM1 were significantly elevated in GDM (**Supplementary Figure 1**). With the rapid development of high-throughput analyses, molecular level targeting therapy has become a major trend in drug development. Molecular docking and molecular dynamic techniques can provide insights into the interactions between molecules and explain the mechanisms of interactions in a visual manner, which have now become important research methods for elucidating biological mechanisms. Additionally, molecular docking and molecular dynamic techniques provide important tools for predicting the binding types and interaction patterns of biomolecular complexes. The mechanisms of action and molecular targets of Wheat-flavored Rehmannia decoction, Astragalus Sijunzi decoction, Radix Astragali and Radix Ophiopogonis decoction, and Shenqi Dihuang decoction for the treatment of GDM are currently largely unknown. To assess whether these traditional Chinese medicines can bind to ferroptosis-related gene loci for preventive and therapeutic effects, the central gene expression proteins were combined as receptors for the downloaded molecular structures of 218 herbal ingredients involved in the four tonics. *Coptis Chinensis* has optimal binding energy when the drug efficacy and drug binding efficacy are combined. Further molecular dynamic simulations demonstrated that SF3B14_2F9D could stably bind and exert potential bioactivity with coptisine, a component of *Coptis Chinensis*, and BABAM1_6H3C could stably bind and exert potential bioactivity with berberine, another component of *Coptis Chinensis*. This result indicates that at the molecular level, *Coptis Chinensis* can bind better to these target proteins, providing a theoretical basis for the treatment of GDM with *Coptis Chinensis*.

There are certain limitations to this study. First, no clear indicator of iron overload was identified, and the ferritin detected in the study was subjected to multiple factors. In addition, Since the retrospective study, pregnant women in the first trimester were mostly examined in the community hospital, so the amount of data and the population was limited. The first trimester and second trimester samples didn’t collected from the same patients. Also, women were all in a pool not differentiating whether they were with pre-pregnancy normal weight, overweight or obese and weight gain during pregnancy due to data limitations. There is some bias in the data, and thus more prospective studies on ferritin and multiple iron-related indicators which need to take into account the pre-pregnancy weight and metabolic condition are needed for more extensive validation. Next, there was limited data available on public databases to screen for relevant genes that met the inclusion criteria; therefore, further validation on a larger scale is anticipated. Only electron microscopy and iron death-related protein alterations were used to verify the occurrence of ferroptosis in GDM placental tissues; hence, further studies are needed to verify the relevance of ferroptosis in the development of GDM. In addition, we selected the optimal PDB IDs and the full selection of active ingredients of herbal tonics currently available on the website for analysis in this article because of technical limitations. Finally, this study lacks validation of SF3B14 and BABAM1 in placental tissues, and related study whether the compound can regulate the expression of ferroptosis related gene. Thus, the genes, proteins, and regulatory mechanisms associated with ferroptosis require further validation.

In conclusion, we verified the elevation of serum ferritin in patients with GDM from a clinical perspective, confirmed the occurrence of ferroptosis in placental tissues in patients with GDM, and proposed an important role of ferroptosis in the pathogenesis of GDM. Next, based on a comprehensive bioinformatics analysis, we screened ferroptosis-related genes with clinical value in GDM, among which SF3B14 and BABAM1 were key genes involved in ferroptosis in the pathogenesis of GDM. Finally, based on the screened proteins corresponding to SF3B14 and BABAM1, we performed molecular docking and molecular dynamic simulations with small molecules that can be bound in clinically proven effective traditional Chinese medicine tonics and proposed the mechanism of action of *Coptis Chinensis* for the treatment of GDM at the molecular level. Thus, our findings provide a theoretical basis for the future clinical application of *Coptis Chinensis*. We have combined a variety of methods to reveal the active ingredients in herbal formulas, and the results might provide new directions for the systematic optimization of TCM formulas for the management and targeted treatment of GDM.

## Data availability statement

The datasets presented in this study can be found in online repositories. The names of the repository/repositories and accession number(s) can be found in the article/**Supplementary Material**.

## Ethics statement

The studies involving humans were approved by Ethics Committee of Shengjing Hospital, China Medical University. The studies were conducted in accordance with the local legislation and institutional requirements. The participants provided their written informed consent to participate in this study. Ethical approval was not required for the studies on animals in accordance with the local legislation and institutional requirements because only commercially available established cell lines were used.

## Author contributions

YuW conceived the study and critically revised the content of this manuscript. YZ, QG, and BL made significant contributions to the study methods, results, and interpretation. YaW was responsible for collecting the patients’ information. QG has done a lot of work on the addition of experiments and the addition of bioinformatics section. All authors contributed to the article and approved the submitted version. 
