# Supplementary figures and images for: Ferroptosis and its potential role in gestational diabetes mellitus: updated evidence from pathogenesis to therapy
Source: Front Endocrinol (Lausanne). 2023 Aug 18;14:1177547. doi: 10.3389/fendo.2023.1177547 (PMC10471987; doi:10.3389/fendo.2023.1177547)

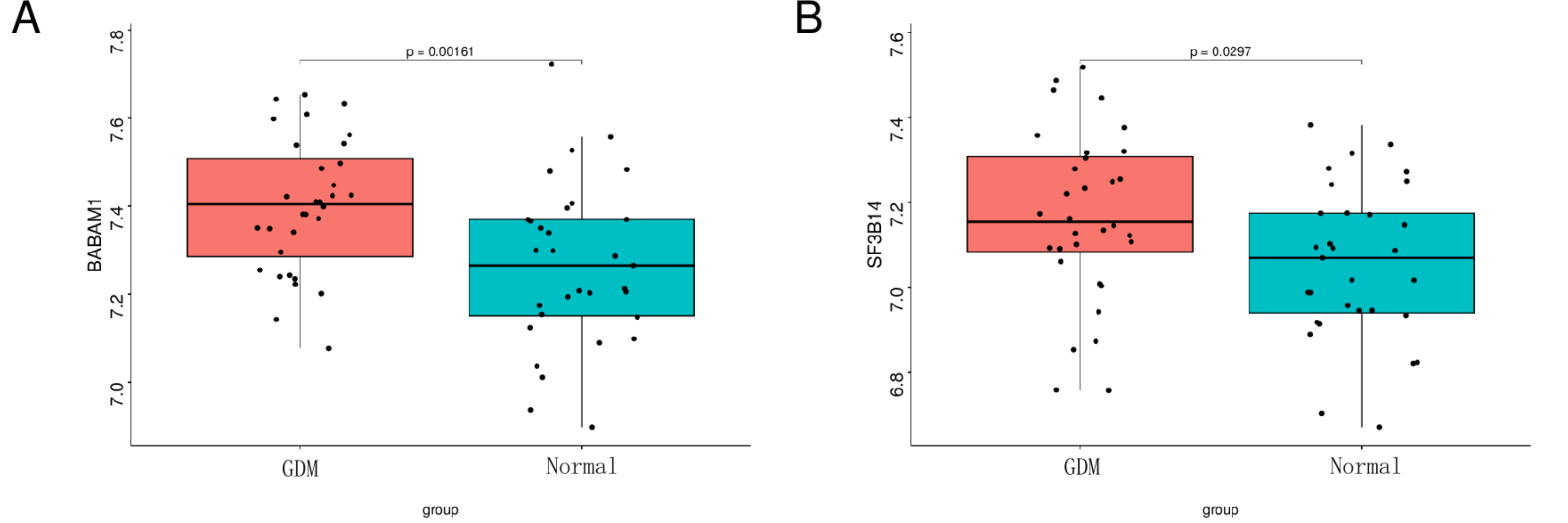

Supplement: Supplementary file 1 [file Image_1.tiff]
